# A Method to Compensate Head Movements for Mobile Eye Tracker Using Invisible Markers

**DOI:** 10.16910/jemr.11.1.2

**Published:** 2018-01-06

**Authors:** Rie Osawa, Susumu Shirayama

**Affiliations:** The University of Tokyo, Japan

**Keywords:** eye movements, gaze behavior, eye tracking, head movements, template matching, invisible marker

## Abstract

Although mobile eye-trackers have wide measurement range of gaze, and high flexibility, it is difficult to judge what a subject is actually looking at based only on obtained coordinates, due to the influence of head movement. In this paper, a method to compensate for head movements while seeing the large screen with mobile eye-tracker is proposed, through the use of NIR-LED markers embedded on the screen. The head movements are compensated by performing template matching on the images of view camera to detect the actual eye position on the screen. As a result of the experiment, the detection rate of template matching was 98.6%, the average distance between the actual eye position and the corrected eye position was approximately 16 pixels for the projected image (1920 x 1080).

## Introduction

Recently, various eye-tracking devices have been introduced to the market, and eye movement analyses is being conducted in many domains. The difference in gaze behavior between novices and experts can be utilized to develop efficient training methods [ 17, 8 ]. Also, the difference when changing color or arrangements of objects can also help for product development or marketing [ 2, 18 ].

There are generally two types of eye-gaze measurement devices, based on the pupil center corneal reflection method which uses near infrared (NIR) illuminators. One is a display installation type, where the NIRs are installed on a PC display to obtain the eye position. The other is a head mounted type, which obtains the coordinates through identifying the gaze position on a viewed image or movie.

In psychological studies, it is common for the subjects’ heads to be fixed, in order to obtain accurate eye-movement measurements. However, in experiments to measure human gaze behavior realistically, restricting the subjects’ head motion is far from the actual conditions, because humans are known to move their heads, consciously or unconsciously. Head motion represents one of the major human physiological behaviors and is essential in daily life [ 4 ], which is why the decision against any motion restriction was made.

For eye tracking, a head-mounted type of device is suitable when considering reality and flexibility. However, there is one problem specific of such devices: the output eye position data is affected by head movements. Sun et al. [12] mention that it is important to remove noise such as head movements from the obtained gaze data in order to detect the degree of concentration of the driver. Therefore, several methods to detect the exact eye position while excluding the effect of head movements have been developed.

In our study, eye tracking is done utilizing a large screen with artificial feature points created by NIR-LEDs which cannot be seen by the naked human eye. Image processing is performed on the image of the view camera in which feature points are recorded, thereby compensating for the head movements. Finally, a method is proposed for automatic output of the exact part of the large screen being viewed by the subject.

## Related Work

There are four major solutions that have been proposed to address the issue of matching eye positions in the view camera with actual eye positions on the screen or to compensate for head movements from entire eye-tracking data.

### Methods based on features in images

 Toyama et al. [ 16 ] proposed using SIFT (Scale-Invariant Feature Transform) features. Points that have high contrast characteristics, or points at the corner, are regarded as key points with highly noticeable features. These are suitable for matching because they are not affected by rotation or scaling. Jensen et al. [ 7 ] applied SIFT features to construct a 3D AOI (Area Of Interest) from eye-tracking data obtained by a head-mounted eye-tracker. Takemura et al. [ 14 ] proposed using PTAM (Parallel Tracking and Mapping) and Chanijani et al. [ 3 ] applied LLAH (Locally Likely Arrangement Hashing) to find feature points. However, these methods require a sufficient number of feature points in the image for accuracy, which may or may not be present depending on the contents of the view camera image.

### Methods with markers

NAC Image Technology [ 11 ] offers a method using AR markers to create artificial feature points, where AR markers captured in the view camera are matched with spatial coordinates. Tomi and Rambli [ 15 ] also proposed using an eye-tracker with an AR application in the calibration of a head-mounted display. Huang and Tan [ 5 ] used circular patterns as markers. However, large markers could influence eye movements due to their size and appearance. Kocejko et al. [ 9 ] proposed an algorithm to compensate for head movements with three cameras (to observe the eye, scene, and head angle) and LED markers. However, objects of view were limited in the monitor as were the movement of the subjects.

### A method with infrared data communication

Tobii Technology offered a solution that uses infrared data communication markers. Eight such markers (approximately 30 mm3) are required for position detection, where each marker communicates with the eye-tracking device and matches the image of the view camera with the respective spatial coordinates. However, the size of such markers could have a significant impact on eye movements. Note that this device is not currently available.

### Methods sensing head movements

Ahlstrom et al. [ 1 ] proposed compensating for head movements using the recorded gaze behaviors in actual driving scenes with a video camera. However, detection of head movement is performed manually for each frame, which is costly. Larsson et al. [ 10 ] applied a gyro, an accelerometer, and a magnetometer. Even though the accuracy has been improved, the synchronization of eye-tracking data and other sensors still remains an issue.

## Proposed Methodology

As mentioned above, methods based on features require a sufficient number of feature points in the image for accuracy. Markers could influence eye movements in the method with AR markers or infrared data communication. In the method sensing head movements, the synchronization of eye-tracking data and other sensors still remains an issue. In our proposed methodology, eye tracking is done utilizing a large screen with artificial feature points created by NIR-LEDs which cannot be seen by the naked human eye. This methodology does not rely on the content of visual stimuli therefore can be applied even when there are not sufficient features there. Furthermore, markers created by NIR-LEDs does not affect eye movements. Image processing is performed on the image of the view camera in which feature points are recorded, thereby compensating for the head movements. Since template matching is automatically performed using image processing, cost is low and processing is fast relatively. Finally, a method is proposed for automatic output of the exact part of the large screen being viewed by the subject.

### Overview of the experimental apparatus

**Figure 1:**
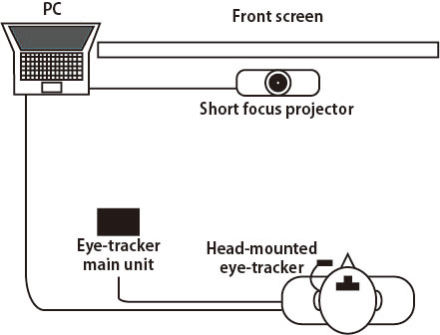
Overview of the experimental apparatus.

Figure 1 illustrates the overview of the experimental apparatus devised to measure the subjects’ gaze behavior while watching the large screen. Figure  illustrates the actual experimental environment.

**Figure 2:**
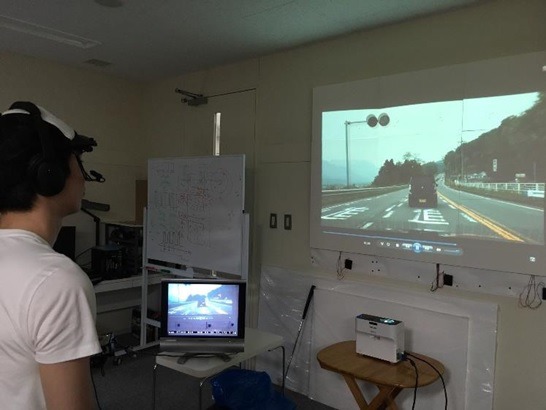
Experimental apparatus for eye tracking.

### Eye-tracking apparatus

**Figure 3:**
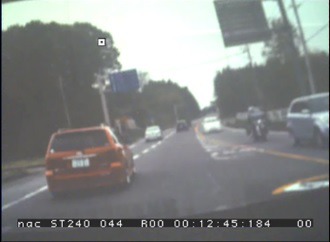
Output image of the view camera.

To record the data of the eye position, we selected NAC Image Technology’s EMR-9 as the eye-tracking device, which includes a view camera attached to the subject’s forehead for video recording. The eye position is indicated by the x–y coordinates in the area recorded by the view camera (Figure 3). Even if the eye position is fixed on a specific item, head movements will cause shifts in the view camera area and the x–y coordinates, leading to difficulties in identifying the target object, as seen in Figure 4a,Figure 4bFigure 4c.

**Figure 4a:**
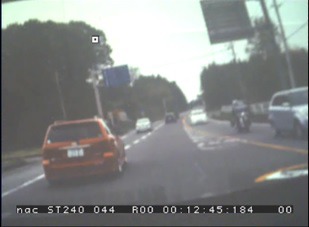
Variations in the view and axis caused by head movements.

**Figure 4b:**
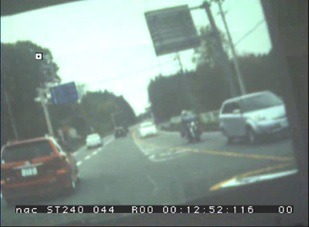
Variations in the view and axis caused by head movements.

**Figure 4c:**
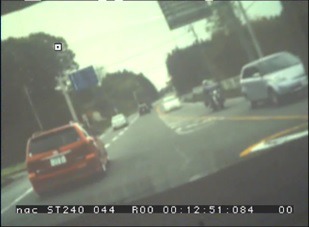
Variations in the view and axis caused by head movements.

### New method using artificial feature points with infrared LED markers

In this paper, a new eye-tracking method is proposed via the creation of artificial feature points made of invisible NIR (near-infrared)-LED markers and image processing. NIR-LEDs are invisible to the human naked eye, therefore reducing their effect on eye tracking despite their presence. At the same time, NIR-LEDs are visible through IR filters, as seen in Figure 5aandFigure 5b. In robot technology, it is popular to use NIR-LEDs to detect locations or to follow target objects [ 13 ]. However, to the authors’ best knowledge, there have been no NIR-LED applications used for eye tracking, which has the potential to enable eye-movement detection even with head movements.

**Figure 5a:**
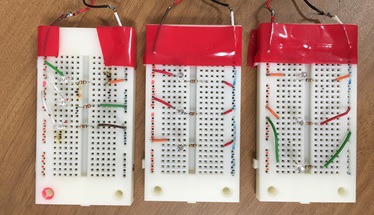
IR markers with the naked eye (left) and through the filter (right).

**Figure 5b:**
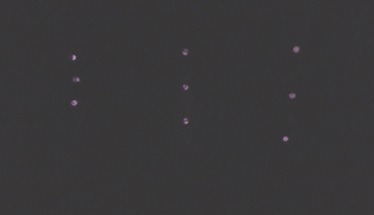
IR markers with the naked eye (left) and through the filter (right).

The view camera with IR filters captures the feature points of NIR-LEDs installed on the projection screen. This image can be used to verify the eye position relative to the NIR-LED feature points, which can then be used to calculate exactly what the subject is looking at on the screen by image processing. We call these invisible NIR-LED markers “IR markers” hereafter.

Image processing is another question that requires attention. SIFT features could be a potential option. However, these methods are not adequate for images of IR markers received through the IR filter, because single NIR-LED IR markers are homogeneous and less characteristic, as shown in the image on the right side of Figure 5. As a countermeasure, several patterns composed of multiple NIR-LEDs have been developed as matching templates, as described below. The overall flow is described later. 

### Patterns of IR markers

IR Marker patterns have been created taking into account the four following conditions. 

1. Patterns should have a sufficient number of features.

2. Patterns should be composed of the smallest number of markers possible.

3. Patterns should be sufficiently differentiable from one another.

4. Patterns should be easily produced.

**Figure 6:**
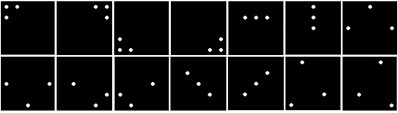
Patterns used for the experiment.

To decide on the exact patterns, the similarities between patterns of filtered IR markers (Figure 6) have been schematically calculated. Taking condition 2 into consideration, a three-point pattern was selected from a 5 × 5 dot matrix for each pattern, which was the best balance to ensure noticeable differentiation. Similarities are calculated by Hu invariant moment algorithm (Hu, 1962).

For a two-dimensional continuous function ƒ(x,y) the moment of order (p + q) is defined as Equation 1.

**Figure:**
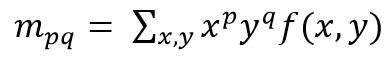


The image moment is the variance value of the pixel centered on the origin of the image. Here, the suffix represents the weight in the axial direction. Subsequently, the centroid is obtained by Equation 2.

**Figure:**
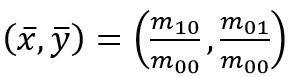


The pixel point (x̅,y̅ ) are the centroid of the image ƒ(x,y). Based on the coordinates of this centroid, the moment considering the centroid is obtained by Equation 3.

**Figure:**
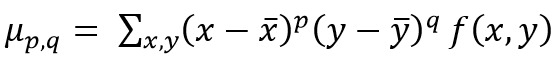


Further, normalize this moment of centroid by Equation 4 to find the normalized centroid.

**Figure:**
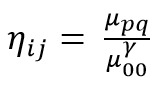


where Equation 5

**Figure:**
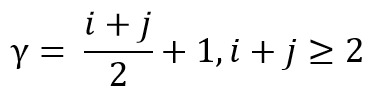


By normalizing, the variance no longer affects the moment value, therefore it is invariant to the scale.

Seven kinds of Hu invariant moment are defined by using the normalized centroid moment, in this study, the moment is calculated by Equation 6

**Figure:**
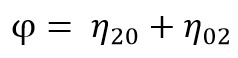


This is the sum of variances in the x-axis direction and the y-axis direction.

Table 1 shows the result of the similarity calculation using the Hu invariant moment.

**Table 1:**
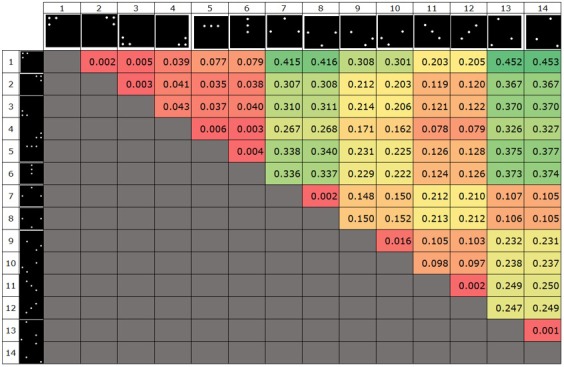
the user interface of ELAN. The software supports multiple synchronized media sources and an arbitrary number of annotation tiers. Videos are blurred to protect participants.

The template images are on the top of the table and the searched images are on the side of the table; lower matching evaluation scores indicate higher similarity and are represented with red cells. The Hu invariant moment allows checks of both rotational and scale invariance; therefore, relevant combinations of patterns with high similarity scores can be calculated.

Based on the findings, several patterns were chosen and created with IR markers. Specifically, NIR-LEDs and resistors were attached to a solder-less breadboard and were mounted onto a polystyrene board. To ensure the high accuracy of the template matching, it was found that twelve patterns were required to be on the board for at least three patterns to be within the view camera at a given time for image processing. The layout of the IR markers was decided based on the similarity results seen in Table 1, and the actual implementation can be seen in Figure 7a and Figure 7b.

**Figure 7a:**
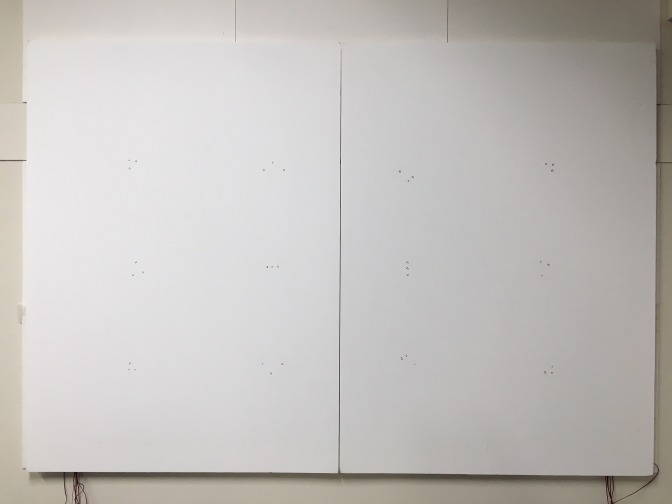
IR Marker-embedded screen as seen with the naked eye (top) and through a filter (bottom).

**Figure 7b:**
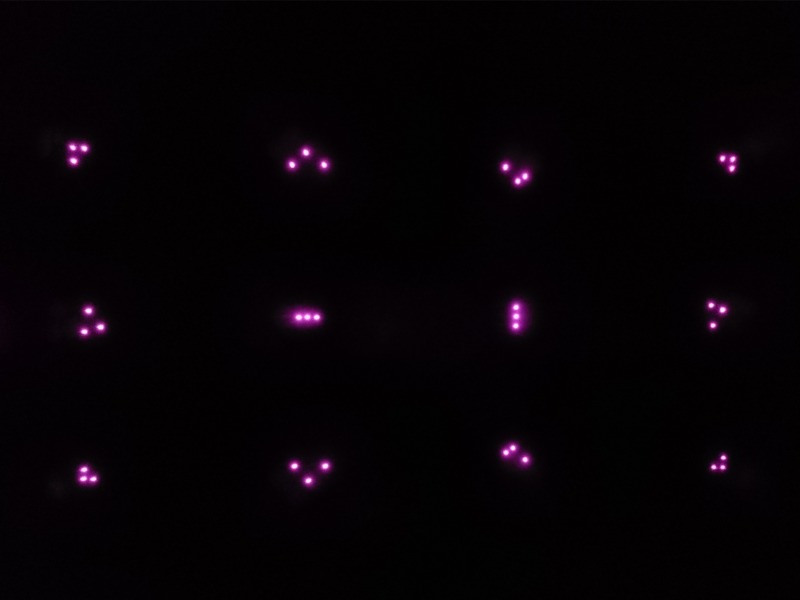
IR Marker-embedded screen as seen with the naked eye (top) and through a filter (bottom).

### Procedure

The operation principle and pattern creating method of NIR-LEDs are described in the previous section. In this section, we will introduce the process and the algorithm of calculating the subject’s view point on the screen, derived from the LED points on the screen and the eye positions.

1. Distortion of the image is caused by the lens of the view camera, therefore calibration is performed for each frame of the obtained movie.

2.Apply template matching on the distortion-corrected images of the view camera to detect the IDs of the IR markers and their coordinates.

**Figure 8:**
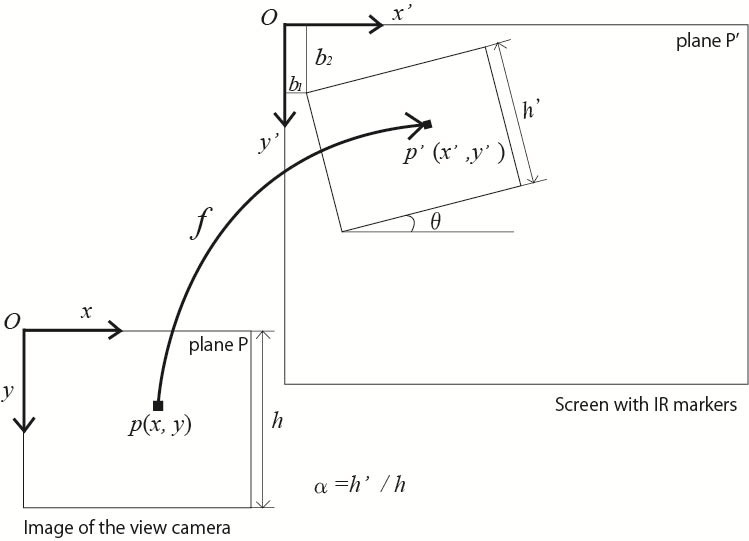
Affine transformation between the image of the view camera and the screen.

3. Detect three points with high matching rates, and obtain their coordinates. In order to calculate the line-of-sight positions on the screen, apply affine transformation to the known coordinates of the markers on the screen.

4. Map the corrected eye coordinates on the image projected on the screen (Figure 8).

5. Output the image or movie with the mapped eye positions (format depends on the visual source).

Affine transformation is used to map coordinates of eye positions in the images of view camera onto the screen. Specifically, scaling is required to adjust the difference in resolution between the view camera and the image projected on the screen, rotation and translation are required to compensate the head movements. Affine transformation is a movement and deformation of a shape that preserves collinearity, including geometric contraction, expansion, dilation, reflection, rotation, shear, similarity transformations, spiral similarities, translation and compositions of them in any combination and sequence.

These transformations for point p(x,y) on a plane to be mapped to point p′(x′,y′) on another plane are expressed as Equation 7.

**Figure:**
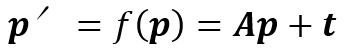


where Equation 8

**Figure:**
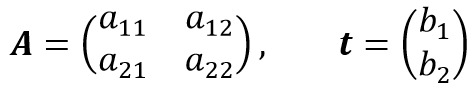


Α represents a linear transformation, and t represents a translation. Scaling can be expressed as Equation 9.

**Figure:**
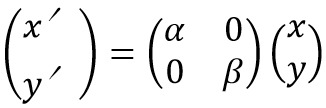


α and β are scale factors of x-axis and y-axis direction respectively. Similarly, rotation can be expressed as Equation 8.

**Figure:**
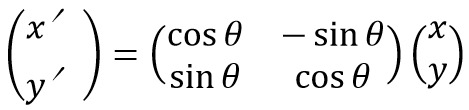


θ is the angle of rotation in the mapped plane. Scaling, rotation and translation are used in this research because distortion caused by the lens of the view camera is calibrated before affine transformation, and scale factor is common to x-axis and y-axis. Therefore, affine transformation matrix required to detect eye positions are obtained by Equation 9.

**Figure:**
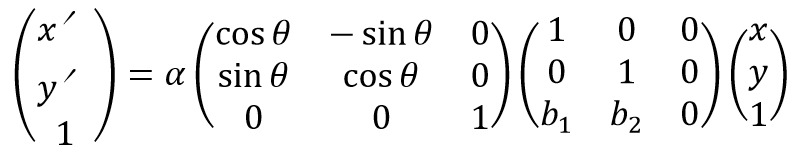


Figure 8 illustrates the image of affine transformation used in our method.

## Verification experiment

### Implementation of the screen for eye tracking

**Figure 9:**
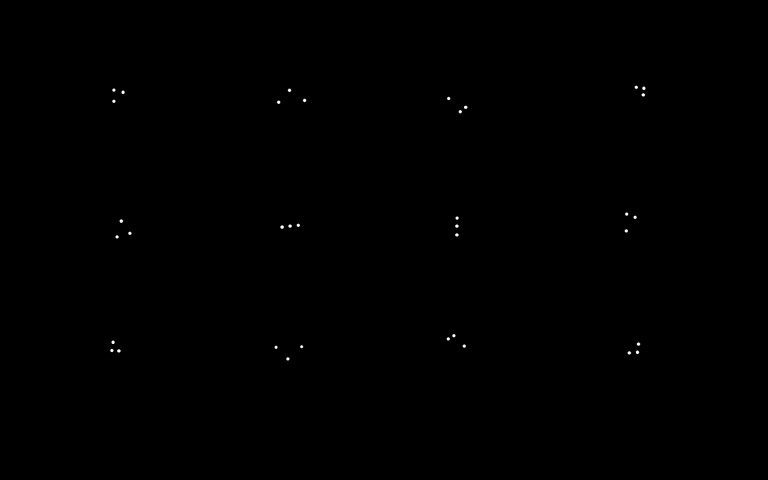
Projected image on the screen for verification experiment.

Verification experiments were conducted to examine the proposed method’s correlation between the eye position, as seen through the view camera, and the actual projected image. Since our method assumes covering the field camera with a filter, the image from the view camera won’t allow detection of what the subject is looking at. In order to verify the results, template matching was conducted by creating a simulated filtered image, by projecting an identical image of that seen on the view camera onto the screen through an IR filter. The image projected on the screen is shown in Figure 9.

### Preliminary experiment

Before conducting template matching of all gaze data, preliminary experiments were conducted to confirm template matching performance. The numbers 1 through 3 were added to the image seen in Figure 9 and projected as shown in Figure 10a and Figure 10b, where the subjects wearing the EMR9 eye tracker were requested to look at them in order. Figure 11 shows an image clipped from the view camera movie during eye-tracking measurements, and Figure 12 represents six template matching results with obtained gaze data.

**Figure 10a:**
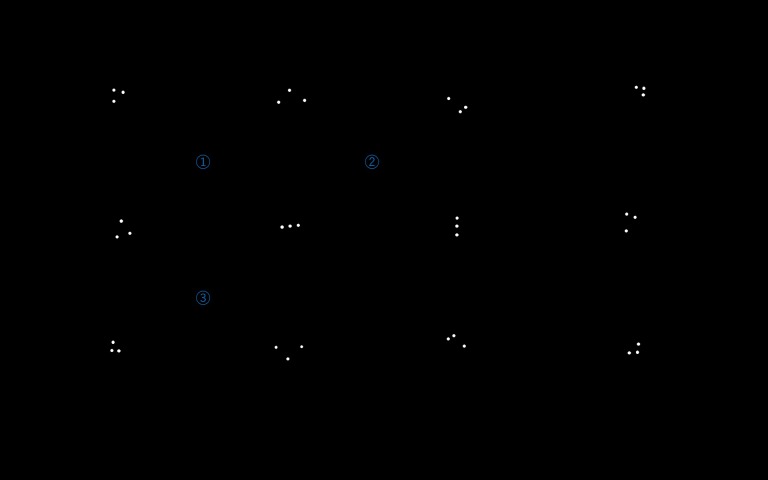
Image source projected on the screen for preliminary experiment (top; yellow circles added for enhancement) and screen with image source projected (bottom).

**Figure 10b:**
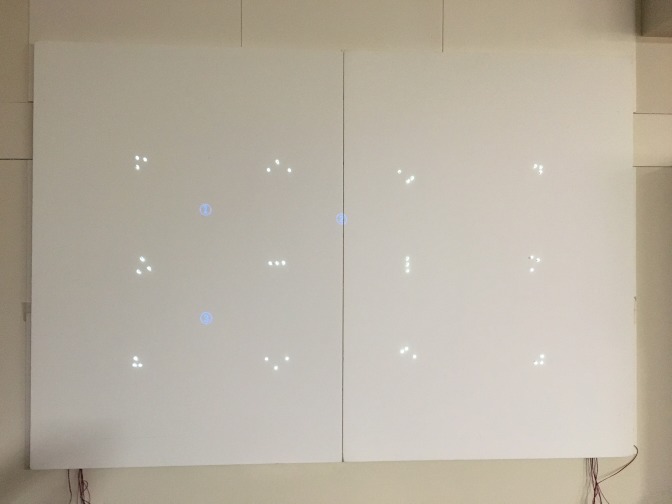
Image source projected on the screen for preliminary experiment (top; yellow circles added for enhancement) and screen with image source projected (bottom).

**Figure 11:**
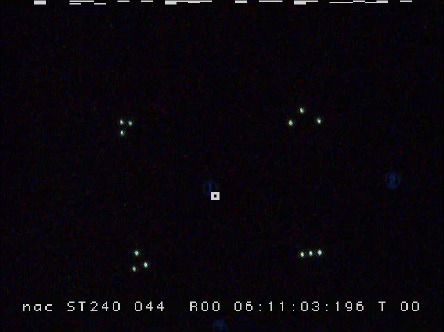
Captured image of the view camera while the subject watching the number 1 on the screen.

**Figure 12:**
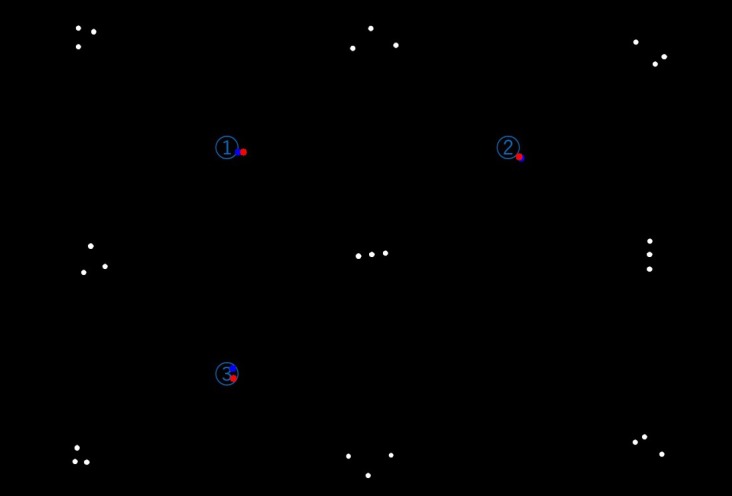
Result of preliminary experiment (enlarged). Eye positions while watching the number 1 through 3 plotted on the projected image through the template matching.

### Result of gaze plot

**Figure 13:**
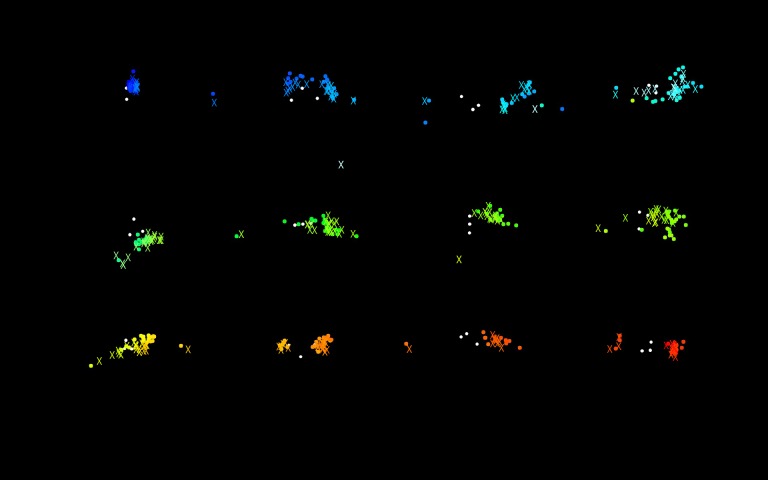
Projected image with eye position mapped (circles: results of template matching, X: results of manual mapping)

Gaze behaviors of the subjects were measured with EMR-9 at 30fps, in a zigzag manner from the upper left marker to the lower right marker of the image shown in Figure 9. Subjects could move their heads freely. To verify template matching performance, Affine transformation was manually conducted based on the template shown in the view camera’s image, and eye positions were mapped onto the projected image. Figure 13 represents template matching results, including a comparison with manually mapped eye points.

Approximately 250 eye points were mapped, where data suggests a very high correlation between template matching and manually conducted mapping results, although some deviation does remain.

**Figure 14:**
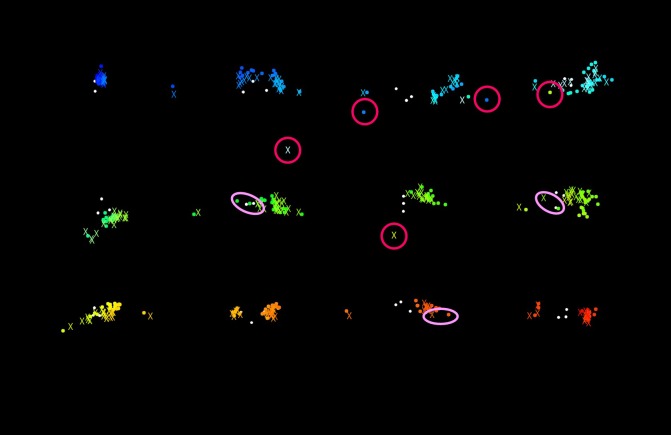
Points containing the errors of template matching (light pink: relatively large gap, dark pink: no correspondence)

**Figure 15:**
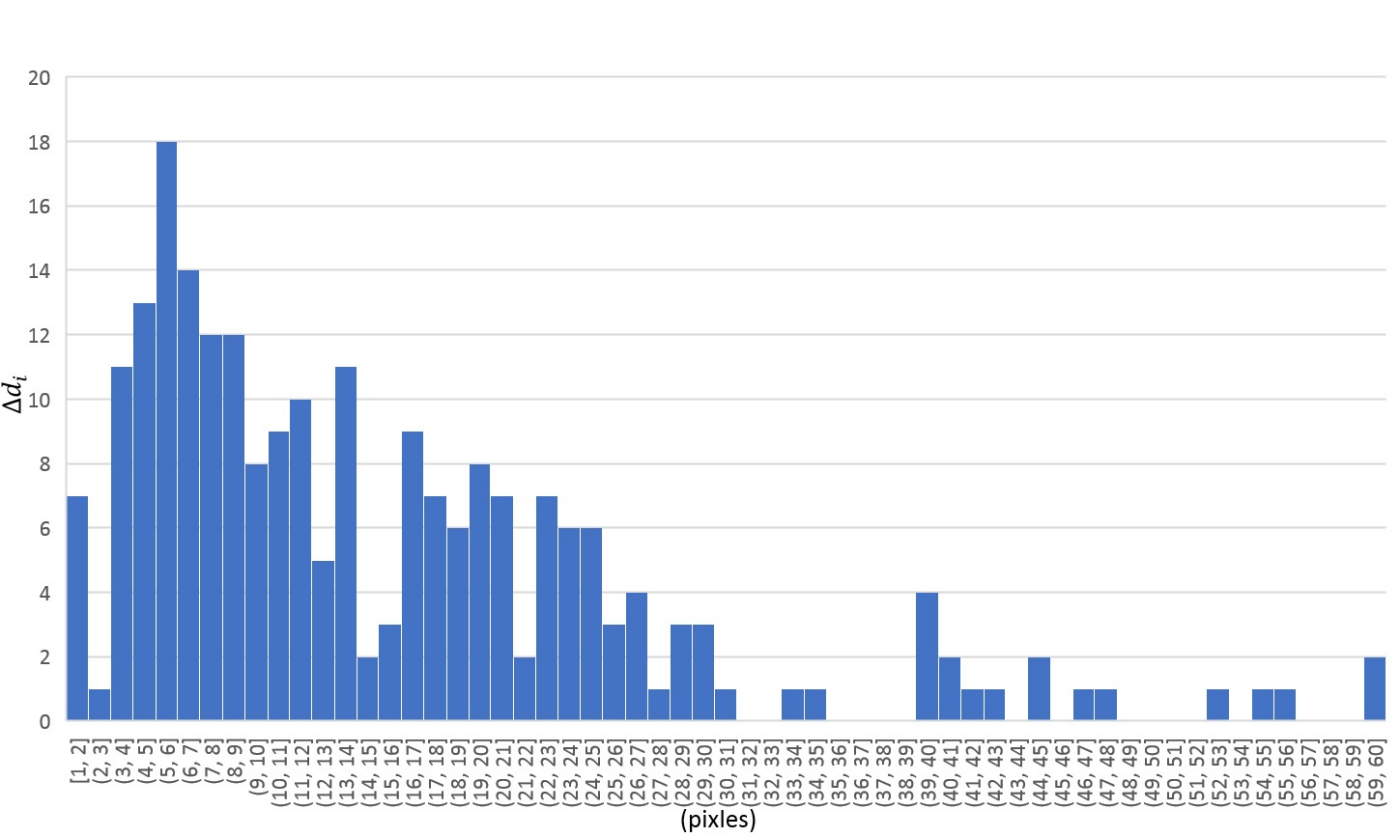
Histogram of distance between the actual eye position and the corrected eye position (∆d_i_ ).

Let ∆d_i_ be the distance between the actual eye position and the corrected eye position, where i denotes the i`th eye points. The detection rate of template matching was 98.6%. Averaged ∆d was 15.9 pixels. Note that the resolution of the projected image was 1920 x 1080 pixels. Points containing detection errors can be seen in Figure 14 and the histogram of ∆d_i_ is represented in Figure 15. More than 90% of ∆d_i_ are within 30 pixels. The main cause of such errors is due to view camera image capture failures, caused by very quick head motions and camera shake, leading to image blur which prevents accurate template matching. However, for example, ∆d_i_ of 30 pixels falls within the range of rear combination lamp of the car shown in the top of Figure 17a, Figure 17b, Figure 17cand Figure 17d (a white circle at point A represents 30 pixels). It can therefore be assumed that our method works in practical use.

**Figure 17a:**
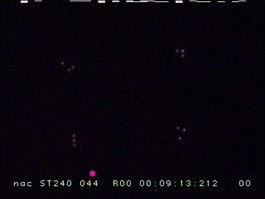
the user interface of ELAN. The software supports multiple synchronized media sources and an arbitrary number of annotation tiers. Videos are blurred to protect participants.

**Figure 17b:**
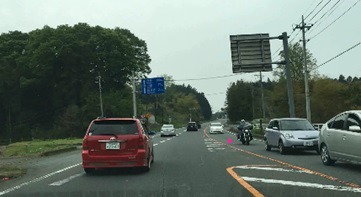
the user interface of ELAN. The software supports multiple synchronized media sources and an arbitrary number of annotation tiers. Videos are blurred to protect participants.

**Figure 17c:**
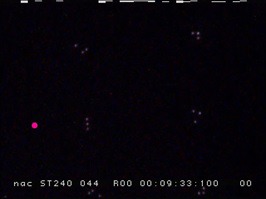
the user interface of ELAN. The software supports multiple synchronized media sources and an arbitrary number of annotation tiers. Videos are blurred to protect participants.

**Figure 17d:**
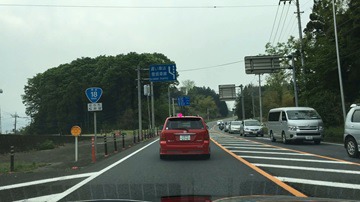
the user interface of ELAN. The software supports multiple synchronized media sources and an arbitrary number of annotation tiers. Videos are blurred to protect participants.

### Gaze plot on the movie

Our method can also be used for gaze measurement while watching a movie, and output the movie with eye point mapped on each frame automatically. Here, a driving video footage taken from the inside of a vehicle while driving was adopted as a visual stimulus. Figure 16a and Figure 16b shows the images clipped from the movie and Figure 17 shows the images of view camera and the corresponding corrected eye positions mapped on the source movie.

**Figure 16a:**
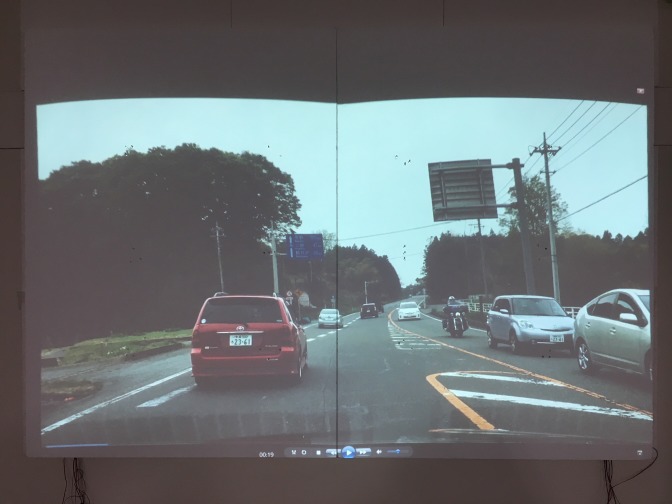
Scene images from a projected movie on the screen.

**Figure 16b:**
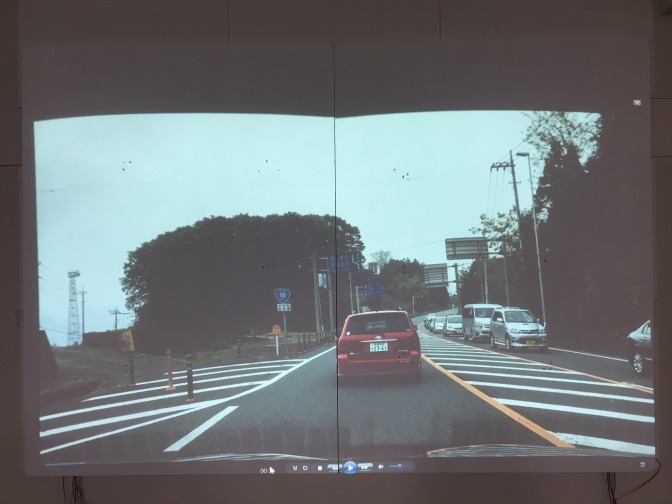
Scene images from a projected movie on the screen.

## Conclusions and Remarks

A new method to compensate for head movement during eye-tracking has been developed, using invisible markers. This will enable higher eye position detection accuracy, which is a problem specific to mobile eye-tracker. However, our methodology has two limitations: First is that the eye tracking is limited on the screen with IR markers embedded. When expanding the range of measurement, it is necessary to add new screens and increase the number of markers newly. Secondly, current apparatus does not allow to confirm the correspondence between the projected image and the eye position in the image of view camera because the view camera is covered with the IR filter. In order to solve this issue, we will add a view camera without filter in the future work.

In addition to the issues to be solved in the future works shown above, error in positioning still remains, due to the error of template matching in some cases, which does have room for improvement for better eye position recognition. Potential solutions to reduce such error include (i) the use of a view camera with higher sensitivity and resolution with shorter exposure time, and (ii) adopting a more robust template matching method. As (i) is less realistic due to the wide use of commercially available eye-trackers with limited performance, a more effective approach would be (ii) through image preprocessing with edge detection as an example.
